# How Habitat Micromodification Influences Gut Microbiota and Diet Composition of Reintroduced Species: Evidence from Endangered Père David’s Deer

**DOI:** 10.3390/microorganisms14010155

**Published:** 2026-01-10

**Authors:** Menglin Sun, Hongyu Yao, Ran Wang, Zeming Zhang, Hong Wu, Dapeng Zhao

**Affiliations:** Tianjin Key Laboratory of Conservation and Utilization of Animal Diversity, College of Life Sciences, Tianjin Normal University, Tianjin 300387, China

**Keywords:** adaptation, non-invasive monitoring, diet, nutrition, gut microbiota

## Abstract

Habitat micromodification poses significant challenges to wildlife, necessitating adaptive responses. This study aimed to investigate how such habitat alterations affect the dietary intake and gut microbiota of Père David’s deer (*Elaphurus davidianus*). A total of 25 fresh fecal samples were collected from Père David’s deer through non-invasive sampling in Tianjin Qilihai Wetland across three distinct phases of habitat micromodification: pre-change (N = 10), under-change (N = 8), and post-change (N = 7). Dietary composition was analyzed via microscopic identification of plant residues, and gut microbiota structure was characterized using 16S rRNA high-throughput sequencing. Results showed that the diet shifted significantly across phases, with 33 plant species from 20 families identified. Meanwhile, the core gut microbiota remained structurally stable. The phyla Firmicutes and Bacteroidota consistently dominated, despite fluctuations in some specific bacterial genera. Functional prediction indicated minimal change in core microbial metabolic pathways. Correlation analysis suggested that key dietary plants were linked to the abundance of specific, functionally relevant microbial taxa. In conclusion, this study demonstrates that the gut microbiota of Père David’s deer exhibits marked resilience to dietary shifts induced by habitat micromodification. This stability is underpinned by functional redundancy within the microbial community and the consistent intake of fibrous plants, representing a key adaptive mechanism. Our findings highlight that integrating non-invasive monitoring of diet and microbiota can effectively assess the adaptive capacity of endangered ungulates to managed habitat change, thereby informing more resilient conservation strategies.

## 1. Introduction

The structural and functional stability of ecosystems is fundamental for maintaining biodiversity and ecological services [1]. As primary consumers, herbivores play a key role in ecosystem energy flow and material cycling. Their population dynamics not only determine their own survival adaptations, but also regulate the efficiency of resource acquisition by secondary consumers and the rate of organic matter conversion by decomposers through top-down trophic cascade effects [2]. While natural disturbances such as floods [3] and earthquakes [4] trigger cyclical fluctuations in ecosystems, human-led habitat transformation often reshapes ecological niche spaces in more significant and lasting ways [5]. Environmental modification of protected areas, as an important means of ecological enhancement, significantly alters microtopographic and hydrological conditions while optimizing ecosystem service functions, thereby affecting plant community structure [6,7], which in turn influences herbivore food source availability and nutrient intake structure through trophic cascade effects [8]. Within the Tianjin Qilihai Wetland, the work of habitat micromodification was carried out in 2023. The main steps were to firstly drain the water from some areas of the wetland to other places, clear the corresponding silt and reinforce its embankment, and then replenish the water to return to the original water level, so as to improve the water ecological environment and establish favorable habitats for wildlife within the Tianjin Qilihai Wetland. The gut microbiota comprises the diverse communities of microorganisms residing within the digestive tracts of animals. These communities have undergone synergistic coevolution with their hosts, developing complex functional relationships through prolonged coexistence. Studies have shown that intestinal microbiota directly participate in the host’s environmental adaptation [9] by regulating key physiological processes such as immune homeostasis [10] and nutritional metabolism [11]. Especially when the composition of food sources changes with the habitat, the dynamic response of the gut microbiota can not only reflect the short-term nutritional stress of the host [12], but also indicate its long-term adaptation potential [13]. Therefore, during the process of habitat transformation, dynamic attention to the composition characteristics of animal food sources and the structure of intestinal flora is helpful for scientifically assessing their health status and survival adaptation process [13,14]. It is notable that feces, as a non-invasive sample [15], can simultaneously provide the composition of food sources [16] and the characteristics of microbiota [12], which provides an ideal entry point for the adaptive monitoring of endangered species. Dietary composition was assessed through microscopic examination of fecal samples. This well-established morphological method could direct identify indigestible plant and animal residues that are ecologically informative. While molecular techniques like high-throughput sequencing offer high resolution for digested matter, microscopic analysis provides complementary data on diet structure and composition that is consistent with classical herbivore foraging studies.

The reintroduction of Père David’s deer (*Elaphurus davidianus*), belonging to the genus *Elaphurus*, as a typical wetland herbivore has important value in its conservation history. It is rated as “Extinct in the Wild (EW)” on the IUCN Red List of Threatened Species [17]. Père David’s deer were once extinct in China due to overhunting and habitat loss in the late 1800s, but were successfully reintroduced in the 1980s through transnational conservation cooperation [18]. Currently, several populations of Père David’s deer have been established in different regions of China, including the Tianjin Qilihai Wetland [19]. Previous studies have shown that although Père David’s deer in different habitats maintain core flora of Firmicutes and Bacteroidetes, their structure and relative abundance are affected by food source seasonality [20] and geographic variability [21]. We hypothesize that targeted habitat micromodification in the Qilihai Wetland drives specific shifts in the winter diet of Père David’s deer, which in turn restructures their gut microbial community, enhancing its adaptation to the modified environment. Therefore, exploring the impact of transformation activities on Père David’s deer living here will help us better explore the relationship between habitat and wildlife. Scientific monitoring of herpetofauna’s adaptive responses to habitat change is of dual significance for maintaining ecosystem integrity and assessing the sustainability of reintroduction projects [22].

In this study, we applied 16S rRNA high-throughput sequencing technology to characterize the gut microbiota, coupled with the analysis of plant components by microscopic observation from Père David’s deer in Tianjin Qilihai Wetland before and after reintroduction of habitat micromodification, in order to investigate the adaptation mechanisms involving dietary composition and gut microbiota. The findings are expected to enhance habitat micromodification and promote the successful reintroduction of Père David’s deer.

## 2. Materials and Methods

### 2.1. Sample Collection and DNA Extraction

In this study, fecal samples were collected from Qilihai Wetland in Tianjin, China, with high activity of Père David’s deer during 2022 to 2024. During this period, Qilihai wetland was undergoing an ecological habitat micromodification. Samples collected before habitat micromodification were designated as the pre-change group, those collected during micromodification as the under-change group, and those post-micromodification as the post-change group. During sampling, sterile cotton swabs and gloves were used to collect approximately 5–8 g (wet weight) of fresh fecal material from the central core of each deposit, minimizing the inclusion of external environmental contaminants. A total of 25 fresh fecal samples were collected during three distinct phases of the habitat modification project: winter 2022 (pre-change group, N = 10), winter 2023 (under-change group, N = 8), and winter 2024 (post-change group, N = 7). To ensure that each sample was from a different individual, we selected samples at certain intervals, and in principle, we chose samples whose quality could simultaneously meet the requirements of molecular sequencing and microscopic examination. As individual Père David’s deer were not marked and they moved freely within the wetland reserve, samples collected in different phases likely originated from different individuals within the resident population. This population-level design assesses the overall state of the deer assemblage in response to habitat changes over time.

Each sample was individually stored in a 50 mL sterilized tube, transported to the laboratory under low-temperature conditions, and preserved at −80 °C in an ultra-low-temperature freezer for further analysis. For DNA extraction, 0.3 g of each fecal sample was weighed, and total DNA was isolated using the TIANamp Stool DNA Kit (Tiangen Biotech Co., Ltd., Beijing, China). DNA concentration and purity were measured with a NanoDrop 2000 spectrophotometer (Thermo Fisher Scientific, Waltham, MA, USA). To confirm host origin, total DNA from each sample was first screened via a species-specific PCR assay.

### 2.2. Analysis of Gut Microbiota

PCR amplification was then performed using the extracted DNA as a template, with primers 515F (5′-GTGCCAGCMGCCGCGG-3′) and 907R (5′-CCGTCAATTCMTTTRAGTTT-3′). PCR was carried out, and the amplified products were detected through 1.0% agarose gel electrophoresis. Then, sequencing was performed on an Illumina HiSeq 2500 platform by Shanghai Majorbio Bio-pharm Technology Co., Ltd. (Shanghai, China).

Raw data were assembled using FLASH software 1.2.11, followed by quality control and filtering of the raw data using Trimmomatic 0.33 and UCHIME 4.2 to obtain valid data, completing the statistical and optimization processes of the raw data. Operational Taxonomic Unit (OTU) clustering analysis was performed at 97% sequence similarity. Taxonomic classification and annotation of the clustered OTU sequences were conducted using the RDP classifier Bayesian algorithm based on the SILVA 138/16s_bacteria reference database. The Shannon index curve plateaus with increasing sequencing depth, indicating that the sample size is sufficient for reliable diversity analysis (Figure S1).

The top five bacterial phyla and genera ranked by relative abundance were defined as dominant phyla and genera. The Kruskal–Wallis test was employed to analyze differences in the top ten most abundant phyla and genera among the pre-change, under-change, and post-change groups in Père David’s deer gut microbiota [23], and the Tukey–Kramer test was selected for the post hoc test [24]. For alpha diversity analysis, Ace, Chao, Shannon, and Simpson indices were calculated, and Wilcoxon’s rank-sum test was applied to assess inter-group differences. To statistically test for significant differences in microbial community structure among sample groups, Analysis of Similarities (ANOSIM) was performed based on weighted unifrac and unweighted unifrac distance algorithms [25]. Functional prediction of gut microbiota across sample groups was preliminarily performed using PICRUSt2 based on the KEGG database. Additionally, Bugbase phenotypic prediction was utilized to analyze the proportional contribution of pathogenic bacteria among different sample groups.

In the statistical analysis results of this study, the significant difference level was defined as 0.01 ≤ *p* < 0.05 and marked with “*” in the relevant graphs. The extremely significant difference level was defined as 0.001 ≤ *p* < 0.01 and marked with “**” in the relevant graph. The super-significant difference level was defined as *p* ≤ 0.001 and marked with “***” in the relevant graph.

### 2.3. Analysis of Dietary Composition

The collected fecal samples were placed in a forced-air drying oven (DHG-9145A, Shanghai Yiheng Scientific Instruments Co., Ltd., Shanghai, China) and dried at 60 °C to a constant weight. For each sample, 0.3 g was ground into powder and immersed in sodium hypochlorite solution (Tianjin Comio Chemical Reagent Co., Ltd., Tianjin, China) until fully submerged. The mixture was stirred with a glass rod to ensure complete immersion and treated for 3–5 h. Temporary slides were prepared to assess cellular morphology clarity. After achieving distinct cellular morphology, the contents were filtered through a 200-mesh sieve, rinsed with distilled water for 2 min and transferred to cleaned Petri dishes. Subsequently, 1–3 drops of 0.01% methylene blue solution were added for staining, followed by rinsing with distilled water to remove excess stain. A dropper was used to transfer cellular fragments onto a glass slide pre-coated with distilled water. Fragments were evenly dispersed using forceps, covered with a coverslip, and prepared for observation.

In this study, plants from Qilihai Wetland were collected and identified according to their structures, such as roots, stems, leaves, flowers and fruits. The plants were also placed in a blast drying oven at 60 °C till reaching a constant weight, and then appropriate samples were taken, fully ground to powder, and treated with sodium hypochlorite solution for 3–5 h. The samples were then rinsed and dyed with 0.01% methylene blue solution. After dyeing, they were rinsed with distilled water to remove the stain and used as a reference for fabricating microstructures.

The temporary plates were observed by a fluorescence microscope (Leica, Wetzlar, Germany). Each sample was observed at 10 × 10 times, and 50 different fields were photographed. The morphology of phytokeratin fragments in each sample was recorded as much as possible, and the recognizable phytokeratin fragments in each field were observed. The species of the plant was identified according to different cell morphological categories. The epidermal cuticle fragment appearing in one field of view was recorded as a single plant, and the number of its occurrences in the field of view was expressed as F, which could be converted to the average density D*_i_* of recognizable plant epidermal fragments of the type of plant in each field of view [12].

$$D_{i}\;=-\ln\left(1-F\;÷\;100\right)$$

D can be converted to a relative density RD.

$$\mathrm{RD}={\;D}_{i}\;÷\;\Sigma D\;\times \;100\%$$

The differences in the dietary compositions of different groups were analyzed based on the Mann–Whitney U test, and SPSS 27.0.1 software was used to complete the analysis.

### 2.4. Potential Correlation Between Dietary Composition and Gut Microbiota

Based on the Spearman ranking coefficient, the potential correlation between the top 3 food species with an RD value of Pere David’s deer and the dominant phylum and genus of gut microbiota was investigated. Origin 2024b software was used to draw a correlation map. The significance level of statistical analysis in this study was set at three levels.

## 3. Results

### 3.1. Results of Gut Microbiota

#### 3.1.1. Basic Sequencing Information

A total of 973,475 optimized sequences were obtained from 25 fecal samples, including 19 phyla, 36 classes, 85 orders, 163 families, 361 genera and 664 species. At 97% similarity, 6419 OTUs were obtained by OTU clustering. Among them, the gut microbiota in the pre-change group comprised 16 phyla, 29 classes, 72 orders, 139 families, 307 genera, and 548 species; the under-change group contained 15 phyla, 26 classes, 61 orders, 110 families, 259 genera, and 478 species; while the post-change group included 15 phyla, 29 classes, 65 orders, 119 families, 268 genera, and 492 species. The common microbiota of the three sample groups included 13 phyla, 21 classes, 49 orders, 89 families, 204 genera, and 361 species. The pre-change group comprised 96 unique species, the under-change group included 52 unique species, and the post-change group comprised 41 unique species (Figure S2).

#### 3.1.2. Species Composition and Differences in Gut Microbiota

**Figure 1:**
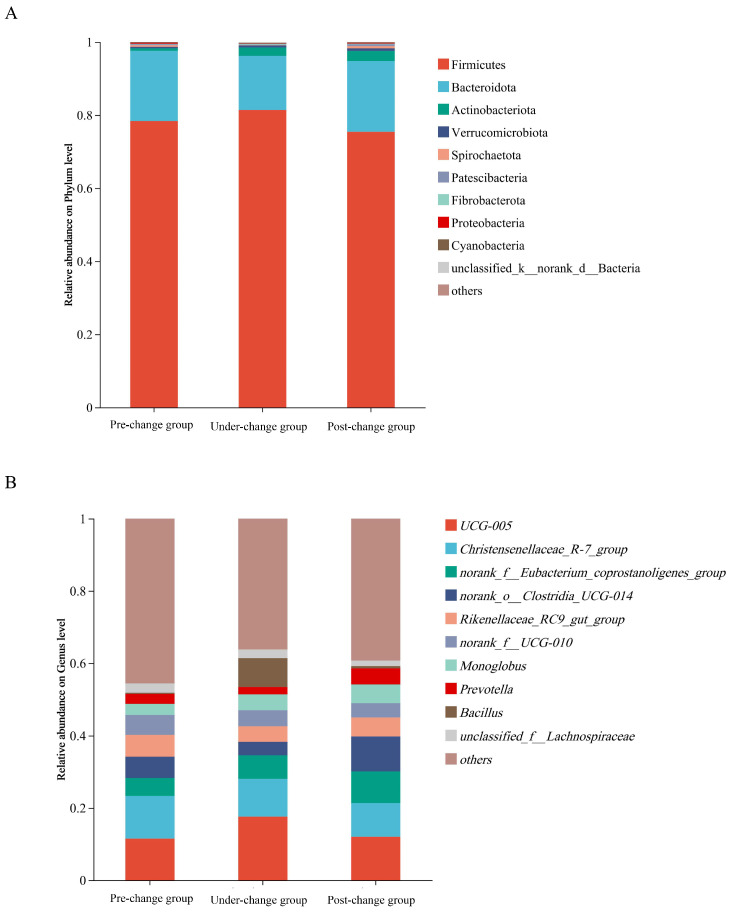
Microbial community composition at phylum and genus levels across different groups. (**A**) The microbial relative abundance at the phylum level among the three experimental groups. (**B**) The microbial relative abundance at the genus level among the three experimental groups.

At the phylum level, the top five most abundant bacterial phyla in the gut microbiota of the pre-change group were Firmicutes (78.37%), Bacteroidota (19.21%), Actinobacteriota (0.69%), Verrucomicrobiota (0.40%), and Spirochaetota (0.45%); in the under-change group, the dominant phyla were Firmicutes (81.38%), Bacteroidota (14.83%), Actinobacteriota (2.29%), Verrucomicrobia (0.54%), and Fibrobacterota (0.26%). For the post-change group, the top five phyla comprised Firmicutes (75.42%), Bacteroidota (19.35%), Actinobacteriota (2.74%), Verrucomicrobiota (0.73%), and Spirochaetota (0.62%) (Figure 1A). The common dominant phyla of three sample groups were Firmicutes, Bacteroidota, Actinobacteriota, and Verrucomicrobiota. The unique dominant bacterial phylum of the pre-change group and post-change group was Spirochaetota, while that of the under-change group was Fibrobacterota.

**Figure 2:**
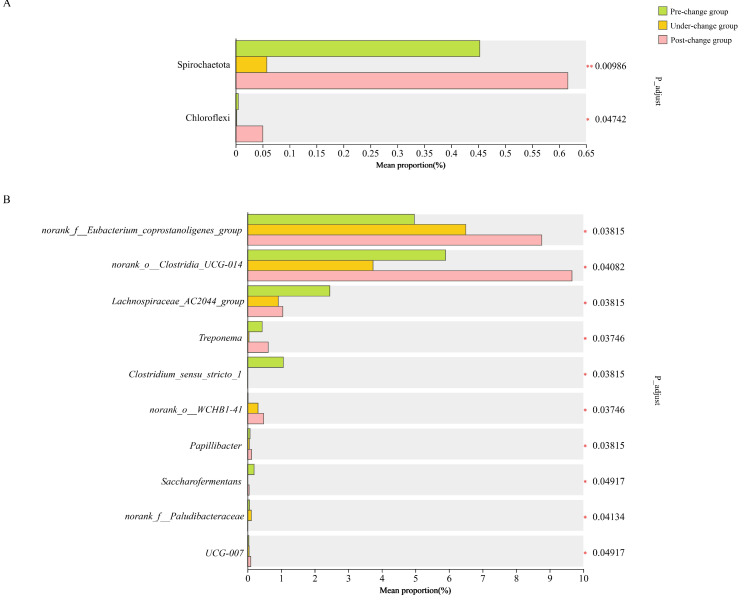
Gut microflora comparison of relative abundance at the phylum level and genus level among different groups. (**A**) Comparison of the relative abundance of gut microbiota at the phylum level among pre-change group, under-change group, and post-change group. (**B**) Gut microflora comparison of relative abundance at the genus level among the three groups. Statistical comparisons of microbial relative abundance among the three groups were conducted using the Kruskal–Wallis test, with false discovery rate (FDR) correction applied for multiple testing. Significance levels are indicated as * 0.01 ≤ *p* ≤ 0.05, ** 0.001 ≤ *p* ≤ 0.01.

The relative abundance of Spirochaetota in the pre-change group was significantly higher than that in the under-change group. The relative abundance of Spirochaetota in the post-change group was significantly higher than that in the under-change group. The relative abundance of Chloroflexi in the post-change group was significantly higher than that in the pre-change group and under-change group (Figure 2A).

At the genus level, the top five relatively abundant bacterial genera in the gut microbiota of the pre-change group were *Christensenellaceae_R-7_group* (11.81%), *UCG-005* (11.50%), *Rikenellaceae_RC9_gut_group* (6.03%), *norank_o__Clostridia_UCG-014* (5.90%), and *norank_f__UCG-010* (5.46%); in the under-change group, the dominant genera comprised *UCG-005* (17.57%), *Christensenellaceae_R-7_group* (10.48%), *Bacillus* (8.01%), *norank_f__Eubacterium_coprostanoligenes_group* (6.50%), and *Monoglobus* (4.39%); and the post-change group exhibited *UCG-005* (12.02%), *norank_o__Clostridia_UCG-014* (9.66%), *Christensenellaceae_R-7_group* (9.31%), *norank_f__Eubacterium_coprostanoligenes_group* (8.76%), and *Rikenellaceae_RC9_gut_group* (5.24%) (Figure 1B, Supplementary Table S1). The common dominant genera of three sample groups were *Christensenellaceae_R-7_group* and *UCG-005*. The unique dominant bacterial genera of the pre-change group were *Rikenellaceae_RC9_gut_group*, *norank_o__Clostridia_UCG-014*, and *norank_f__UCG-010*, while those of the under-change group were *Bacillus*, *norank_f__Eubacterium_coprostanoligenes_group,* and *Monoglobus*, and the unique dominant bacterial genera of the post-change group were *norank_o__Clostridia_UCG-014*, *norank_f__Eubacterium_coprostanoligenes_group,* and *Rikenellaceae_RC9_gut_group*.

Comparing the pre-change group with the under-change group, the relative abundance of *Treponema* and *Clostridium_sensu_stricto_1* in the pre-change group was significantly higher than that in the under-change group, and *Lachnospiraceae_AC2044_group* was significantly higher than that in the under-change group. Comparing the pre-change group with the post-change group, the relative abundance of *Clostridium_sensu_stricto_1* in the pre-change group was significantly higher than that in the post-change group, and *Lachnospiraceae_AC2044_group* was extremely significantly higher than that in the post-change group. The relative abundance of *norank_o__WCHB1-41* in the post-change group was significantly higher than that in the pre-change group, and *norank_f__Eubacterium_coprostanoligenes_group* and *Papillibacter* were extremely significantly higher than those in the pre-change group. Comparing the under-change group with the post-change group, the relative abundance of *norank_o__Clostridia_UCG-014* in the post-change group was significantly higher than that in the under-change group; *Treponema* and *UCG-007* were extremely significantly higher than those in the under-change group; and *Papillibacter* was also significantly higher than that in the under-change group (Figure 2B).

Species composition was compared at the class, order, and family levels, respectively (Figure S3).

#### 3.1.3. Alpha Diversity and Beta Diversity Analysis

**Figure 3:**
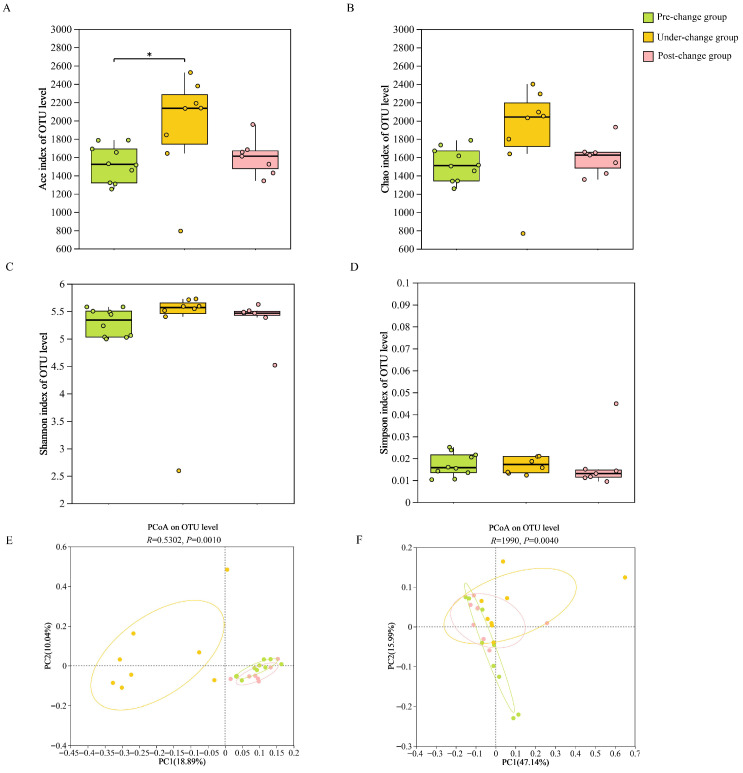
Comparative analysis of gut microbial diversity across pre-change, under-change, and post-change experimental groups. (**A**) Alpha diversity measured using the ACE index; (**B**) alpha diversity measured using the Chao1 index; (**C**) alpha diversity measured using the Shannon index; (**D**) alpha diversity measured using the Simpson index; (**E**) Principal Coordinates Analysis (PCoA) constructed based on the unweighted UniFrac distance matrix; (**F**) PCoA constructed based on the weighted UniFrac distance matrix. Group comparisons were performed using one-way ANOVA (*p* < 0.01). Significance levels are indicated as * 0.01 ≤ *p* < 0.05.

Analysis of alpha diversity revealed significantly greater gut microbiota richness in the under-change group versus the pre-change group (Ace index, *p* < 0.05). Although the under-change group exhibited the highest richness among all groups, microbial diversity indices showed a contrasting trend (post-change > pre-change > under-change), which did not reach statistical significance (Figure 3, Supplementary Table S2). Beta diversity analysis revealed that samples within each group clustered relatively closely, while significant differences in beta diversity were observed between the different groups based on weighted UniFrac (Figure 3E) and unweighted UniFrac (Figure 3F).

#### 3.1.4. Prediction of Gut Microbiota Function

**Figure 4:**
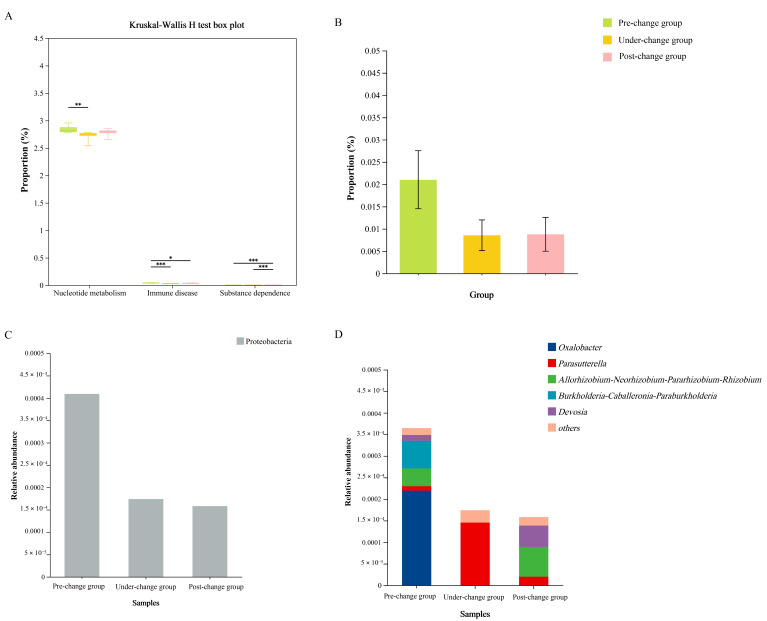
Pathogenicity prediction and function prediction analysis. (**A**) The difference comparisons of functional pathways based on the Kruskal–Wallis H test; (**B**) the proportion of pathogenic bacteria among the three groups; (**C**) the composition of pathogenic bacteria at the phylum level; (**D**) the composition of pathogenic bacteria at the genus level. Significance levels are indicated as * 0.01 ≤*p* < 0.05, ** 0.001 ≤*p* < 0.01 and *** *p* < 0.001.

Based on the KEGG database, only 3 out of 46 secondary metabolic pathways of the gut microbiota showed significant differences among the three groups (Figures S5–S7). In the pre-change group, the relative abundance of nucleotide metabolism was extremely significantly higher than that in the under-change group. The relative abundance of immune disease in the pre-change group was significantly higher than in the post-change group and extremely significantly higher than in the under-change group. In the post-change group, the relative abundance of substance dependence was extremely significantly higher compared to both the pre-change group and the under-change group (Figure 4A).

#### 3.1.5. Pathogenicity Analysis of Gut Microbiota

Pathogenic potential prediction using BugBase phenotype profiling indicated a higher relative abundance of potentially pathogenic bacteria in the post-change group compared to the pre-change and under-change groups; however, no significant inter-group differences were detected (*p* > 0.05) (Figure 4B).

At the phylum level, potentially pathogenic bacteria across all groups primarily belonged to Proteobacteria (Figure 4C). Genus-level analysis revealed distinct compositional differences. The pre-change group was dominated by *Oxalobacter*, the under-change group primarily contained *Parasutterella*, while the post-change group featured *Devosia* and the *Allorhizobium-Neorhizobium-Pararhizobium-Rhizobium* group (Figure 4D).

### 3.2. Dietary Composition

In this study, a total of 1250 photos were taken from 25 fecal samples, and a total of 33 species of Père David’s deer foraging plant species from 32 genera and 20 families were identified (Table 1).

The results of fecal microhistological analysis showed that the pre-change group consumed 29 species of 18 families and 28 genera. Among them, the top five species were *Echinochloa crusgalli* (33.76%), *Phragmites australis* (16.34%), *Setaria viridis* (5.77%), *Acalypha australis* (5.37%), and *Cynanchum chinense* (5.30%). At the family level, the main species was Poaceae (60.23%). Comparative analysis revealed significant variations in plant species from different groups. A total of 27 species of plants from 26 genera and 16 families were collected from the under-change group; the top five species were *Ipomoea purpurea* (30.22%), *Humulus scandens* (11.23%), *Phragmites australis* (10.85%), *Cynanchum chinense* (7.19%), and *Echinochloa crusgalli* (4.25%). Convolvulaceae (30.22%) and Poaceae (24.95%) were the main plants at the family level. The post-change group consumed 28 species of plants from 27 genera and 17 families. The top five species were *Euonymus japonicus* (26.77%), *Phragmites australis* (16.74%), *Cynanchum chinense* (8.21%), *Humulus scandens* (6.38%), and *Ipomoea purpurea* (7.18%). At the family level, the main species were Poaceae (27.27%) and Celastraceae (26.77%). The main feeding plants shared by the three groups included *Phragmites australis* and *Cynanchum chinense*, with Poaceae represented at the family level. The unique major food in the pre-change group included *Echinochloa crusgalli*, *Setaria viridis*, and *Acalypha australis*; the under-change group specifically consumed *Ipomoea purpurea*, *Humulus scandens*, and *Echinochloa crusgalli*, with Convolvulaceae and Cannabaceae represented at the family level; the post-change group uniquely utilized *Euonymus japonicus*, *Humulus scandens*, and *Ipomoea purpurea*, with Celastraceae, Apocynaceae, and Cannabaceae observed at the family level (Table 1).

Based on the Mann–Whitney U test, the top five foods in each group were selected to analyze the significance of differences among the groups. The results showed that in the pre-change group, the RD value of *Phragmites australis* (*p* = 0.034) was significantly higher than that in the under-change group, and the RD value of *Echinochloa crusgalli* (*p* = 0.00005) was significantly higher than that in the under-change group, while the RD value of *Acalypha australis* (*p* = 0.006) was extremely significantly higher than that in the under-change group. The RD value of *Echinochloa crusgalli* (*p* = 0.00005) was significantly higher compared to the under-change group. Conversely, the RD values of *Humulus scandens* (*p* = 0.0009) and *Ipomoea purpurea* (*p* = 0.00005) were significantly lower than those in the under-change group. In the comparisons between the pre-change group and post-change group, the RD value of *Acalypha australis* (*p* = 0.028) was significantly higher in the pre-change group, while *Echinochloa crusgalli* (*p* = 0.0006) exhibited a super significantly higher RD value compared to the post-change group. The RD values of *Euonymus japonicus* (*p* = 0.0001) were super significantly lower than those in the post-change group. For the under-change group versus post-change group, the RD value of *Humulus scandens* (*p* = 0.021) in the under-change group was significantly higher than that in the post-change group, and the RD value of *Ipomoea purpurea* (*p* = 0.0012) in the under-change group was extremely significantly higher than that in the post-change group, whereas the RD value of *Euonymus japonicus* (*p* = 0.0004) was significantly lower than that in the under-change group.

### 3.3. Diet–Microbiota Correlations Across Habitat Micromodification Phases

Spearman correlation analysis was performed to evaluate associations between gut microbiota (phylum level and genus level) and food plants, and significant associations between dominant dietary plants and gut microbial taxa were identified in all three phases, indicating plant-specific modulation of the microbiota.

**Figure 5:**
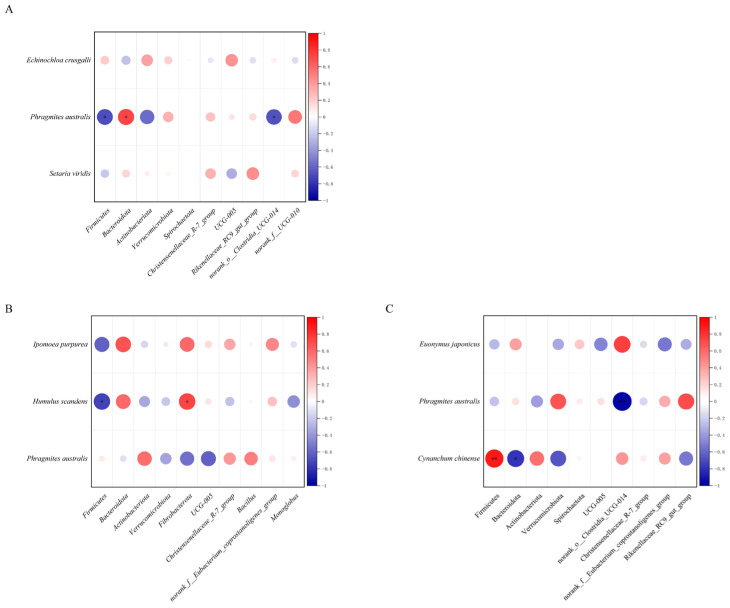
Diet–microbiota covariance networks. Spearman rank correlation-based networks show how associations between the primary diet (top 3 plant species by RD value) and the dominant gut microbial taxa in Pere David’s deer changed after habitat micromodification. (**A**) The pre-change network shows baseline associations prior to habitat micromodification. (**B**) The under-change network reveals associations during habitat micromodification. (**C**) The post-change network illustrates diet–microbe association patterns established after habitat micromodification. Significance levels are indicated as * 0.01 ≤*p* < 0.05, ** 0.001 ≤*p* < 0.01 and *** *p* < 0.001. The size of each circle corresponds to the absolute value of the correlation value.

In the pre-change group, the RD value of *Phragmites australis* was significantly positively correlated with the relative abundance of Bacteroidota (*p* = 0.019) and significantly negatively correlated with the relative abundances of Firmicutes (*p* = 0.025) and n*orank_o__Clostridia_UCG-014* (*p* = 0.033) (Figure 5A).

In the under-change group, the RD value of *Humulus scandens* was significantly positively correlated with the relative abundance of Fibrobacterota (*p* = 0.037) and significantly negatively correlated with Firmicutes (*p* = 0.037) (Figure 5B).

In the post-change group, the RD value of *Phragmites australis* was significantly negatively correlated with the relative abundance of *norank_o__Clostridia_UCG-014* (*p* = 4.5 × 10^−4^). The RD value of *Cynanchum chinense* was extremely significantly positively correlated with the relative abundance of Firmcutes (*p* = 0.0068) and significantly negatively correlated with Bacteroidota (*p* = 0.036) (Figure 5C).

## 4. Discussion

This study compared the dietary habits and gut microbiota characteristics of Père David’s deer across three phases of habitat micromodification (pre-change, under-change, and post-change). It revealed a key paradoxical phenomenon that despite significant shifts in the primary food composition among the three groups corresponding to these habitat phases, the structural composition of the gut microbiota remained remarkably stable. This suggests that the Père David’s deer gut microbiome may buffer dietary perturbations induced by habitat micromodification through specific adaptive mechanisms.

### 4.1. Structural Conservation of Gut Microbiota Amidst Habitat Alteration

This study revealed significant stability in the core structure of the Père David’s deer gut microbiota across the pre-change, under-change, and post-change habitat micromodification phases. The top four dominant bacterial phyla consistently identified in fecal samples from all three periods were Firmicutes, Bacteroidota, Actinobacteria, and Verrucomicrobia. Firmicutes and Bacteroidota together constitute the absolute dominant bacterial phylum (97.58% in pre-change group, 96.21% in the under-change group, 94.77% in the post-change group). This finding aligns with results from studies on Père David’s deer from different geographical populations [11,20,21,26] and other cervids, such as white-lipped deer (*Cervus albirostris*) [27] and red deer (*Cervus elaphus*) [28]. Despite inhabiting diverse ecosystems, these species’ gut microbiomes were consistently dominated by Firmicutes and Bacteroidota. The herbivorous nature of ruminants necessitates gut microbes for digesting key nutrients [29]. Many Firmicutes bacteria possess functions for degrading cellulose and other carbohydrates [30], while Bacteroidota primarily degrade starches, polysaccharides, and proteins [31,32]. Together, they facilitate the digestion of various plant-based foods, providing energy to the host and forming a crucial microbial foundation for ruminants to adapt to diverse habitats. Actinobacteria are common soil microbes [33] but are also present in the rumen, actively influencing host digestion [34]. Verrucomicrobia members typically possess hemicellulose-degrading capabilities [35] and participate in host food digestion. Therefore, this study further confirms that the core microbial structure within the Père David’s deer gut microbiome exhibits strong ecological resilience, even during anthropogenic habitat micromodification. This resilience, potentially evolved through long-term adaptation to buffer environmental pressures, provides additional evidence for understanding the evolutionary conservation and ecological adaptation mechanisms of cervid gut microbiomes.

Beyond these four phyla, Fibrobacterota ranked fifth in the under-change group. Fibrobacterota, containing only the genus Fibrobacter, possesses genes encoding cellulases and is a typical cellulose-degrading bacterium [36,37]. Studies indicate its key role as a lignocellulose degrader in the rumen [35]; its colonization in the Père David’s deer gut likely aids in digesting dietary cellulose for energy. Spirochaetota ranked fifth in the pre-change and post-change groups. Some bacteria within this phylum have been found to possess pectin and hemicellulose-degrading functions in the bovine rumen [35], suggesting their colonization may assist Père David’s deer in digesting hemicellulose and pectin.

At the genus level, shifts occurred in the dominant genera across the three groups, potentially reflecting precise metabolic regulation through functional genus turnover. *UCG-005* and *Christensenellaceae_R-7_group* were the predominant genera shared by all three groups. *UCG-005* belongs to the Ruminococcaceae family, members of which secrete cellulases, hemicellulases, and pectinases to break down plant cell walls. *UCG-005* specifically produces acetate and short-chain fatty acids (SCFAs) to provide energy for the host and is associated with increased intestinal nutrient absorption [38,39,40]. *Christensenellaceae_R-7_group*, a common probiotic in animal guts, regulates immune function and maintains gastrointestinal homeostasis [41]. It also degrades cellulose and hemicellulose in food [42] and plays important roles in amino acid and lipid metabolism [43]. The colonization of these two genera, adapted to the host’s herbivorous nature, effectively assists in digesting dietary cellulose. *Rikenellaceae_RC9_gut_group* also possesses hemicellulose-degrading capacity [44] and is associated with body fat and volatile fatty acid production [45]. Both *Christensenellaceae_R-7_group* and *Rikenellaceae_RC9_gut_group* play critical roles in carbohydrate digestion and absorption within the rumen of ruminants [46]. The relative abundance of *Rikenellaceae_RC9_gut_group* ranked within the top five in the pre-change (6.03%) and post-change (5.24%) groups. Although it dropped out of the top five in the under-change group (4.30%), its abundance did not differ substantially from the other groups.

Notably, *Bacillus* emerged as a dominant genus (8.01%) only in the under-change group, whereas its abundance was minimal in the pre-change (0.34%) and post-change (0.67%) groups. *Bacillus* is ubiquitous in soil and on plant surfaces [47]. Its surge in abundance likely stems from Père David’s deer encountering disturbed soil or newly planted vegetation during habitat micromodification, indicating short-term colonization by environmental microbes. Furthermore, some *Bacillus* members exhibit potential for degrading complex organic matter [48,49], and cellulose-degrading *Bacillus* strains have been isolated from Père David’s deer feces [50]. Thus, the increased *Bacillus* content in the under-change group may assist the host in adapting to dietary shifts during this phase.

The genus *norank_o__Clostridia_UCG-014* increased significantly post-change, rising to the second rank (9.66%), forming a functional synergy with *Eubacterium_coprostanoligenes_group* (8.76% post-change). The former belongs to the Clostridiaceae family and participates in carbohydrate fermentation [51]. The latter is involved in bile acid and cholesterol transformation in the gut; some members produce butyrate, playing important roles in energy homeostasis, suppressing gut inflammation, and immune regulation [52]. Their increased abundance may reflect a greater intake of plant sterols and complex polysaccharides in the post-change habitat. The microbiota potentially aids host adaptation to the new diet by enhancing energy extraction and anti-inflammatory metabolism.

Despite significant fluctuations in the abundance of some genera alongside dietary changes, only 3 of 46 KEGG level 2 metabolic pathways showed significant differences. This suggests high functional redundancy within the microbial community [53], where core metabolic functions may be maintained cooperatively by different genera, resulting in overall stable metabolic function. Pathogenic potential prediction indicated that pathogenic bacteria constituted a very minor proportion across all groups, implying that habitat micromodification did not introduce significant pathogenic risks. Père David’s deer thus demonstrate adaptability to a certain level of anthropogenic disturbance.

### 4.2. Diet–Microbiota Dynamics in Père David’s Deer During Habitat Micromodification

While habitat micromodification likely altered food availability for Père David’s deer to some degree, the animals exhibited strong adaptability through dynamic adjustments in dietary composition. Fecal analysis revealed distinct foraging strategies across the habitat phases. The pre-change group primarily consumed Poaceae plants, with barnyard grass (*Echinochloa crus-galli*) and reed (*Phragmites australis*) constituting over 60% of their diet. For northern ruminants facing limited winter food resources, efficient utilization of fiber-rich foods is crucial [54]. Reeds and other Poaceae plants are rich in cellulose [55,56], meeting Père David’s deer’s basic energy demands during this period. Foraging on widely distributed reeds also aligns with the “time minimization strategy” [57,58], aiding Père David’s deer in efficiently utilizing limited resources during the cold season.

During the modification phase (under-change group), Convolvulaceae plants (field bindweed, *Convolvulus arvensis* 30.22%) and Cannabaceae plants (common hop, *Humulus lupulus* 11.23%) became major food sources. The post-change group reverted to Poaceae dominance (27.27%), but Celastraceae plants (Japanese spindle, *Euonymus japonicus*) significantly increased (26.77%). Previous studies showed that Père David’s deer in Hubei Shishou Milu National Nature Reserve primarily consumed C3 plants in autumn and winter [59], while Père David’s deer in Beijing Nanhaizi Milu Park consumed Salix and Amorpha in winter [17]. This dietary variation indicates that Père David’s deer are not strictly dependent on a few specific plants but exhibit considerable flexibility in selecting food based on environmental resource availability. This dietary plasticity provides a crucial ecological foundation for Père David’s deer reintroduction and population expansion. Vegetation composition can vary greatly across potential reintroduction sites, and Père David’s deer’s ability to utilize diverse food sources reduces reliance on specific plants, enhancing reintroduction success. Furthermore, this adaptability helps maintain population stability in the face of habitat changes induced by climate change or human disturbance.

Microscopic analysis of epidermal fragments identified residues from a total of 33 plant species. For quantitative assessment, we focused on the dominant dietary components, which included reed (*Phragmites australis*) [56] and Chinese silk vine (*Cynanchum chinense*). Correlation analyses revealed dynamic associations between specific dietary plants and gut microbiota. Notably, reed (*Phragmites australis*), a dominant shared food source in both the pre- and post-change phases, exhibited consistent phylum-level response patterns: its relative density (RD) correlated negatively with Firmicutes and positively with Bacteroidota across both phases. This carries significant functional implications: while both phyla ferment dietary fibers and fats to produce short-chain fatty acids (SCFAs), Bacteroidota possess superior fiber-degrading capacity [60,61]. This aligns with findings that carbohydrate intake specifically promotes Bacteroidota proliferation and elevates SCFA production, indicating reed’s role in directionally regulating microbiota function as a high-fiber plant.

At the genus level, key functional taxa demonstrated metabolic plasticity. In the pre-change group, the RD of reed showed a significant positive correlation with the relative abundance of the fiber-degrading genus *norank_o__Clostridia_UCG-014* (Clostridiaceae family). Remarkably, in the post-change phase, the RD of Chinese silk vine (*Cynanchum chinense*) also correlated positively with this same genus. Given that Clostridia are established degraders of plant cell wall polysaccharides [62], we propose that this taxon primarily assisted in cellulose breakdown from reeds during the pre-change phase. Following the dietary shift, it likely adapted its enzymatic machinery to metabolize specific chemical constituents in *C. chinense* (e.g., C21 steroids, flavonoids).

The overarching adaptive mechanism reflects a co-evolutionary strategy. Père David’s deer mitigated habitat disturbance through a dual buffering system. At the behavioral ecology level, their dietary flexibility enables dynamic selection of available vegetation resources. At the microbial level, the structural stability of the core microbiota (e.g., Firmicutes/Bacteroidetes ratio) and the plasticity of functional genera (e.g., metabolic shifts in Clostridium) jointly maintain digestive and metabolic homeostasis. This synergy between host behavior and microbial function resonates with nutrient-adaptive mechanisms observed in birds and elephant populations, underscoring the critical role of the gut microbiome in wildlife adaptation to environmental change.

Critically, while our findings demonstrate significant Père David’s deer resilience to moderate anthropogenic disturbance, ongoing monitoring of disturbance thresholds is imperative. During the mid-stage of the modification, the proportion of pathogenic bacteria was the lowest, which may indicate that the current habitat micromodification has been highly effective, improving the diet and living conditions of Père David’s deer, promoting the growth of beneficial bacterial communities, and inhibiting the proportion of pathogenic bacteria. This study employed a population-level sampling design, where fecal samples across different time phases likely came from different individuals. While this provides valuable insights into the overall response of the  Père David’s deer assemblage to habitat micromodification, it precludes definitive conclusions about the inter-individual temporal stability of the gut microbiota. Future studies utilizing mark-recapture techniques or continuous GPS tracking of collared individuals would be ideal for disentangling individual-level plasticity from population-level shifts.

## 5. Conclusions

As a herbivorous species, the gut microbiota of Père David’s deer plays a critical role in adapting to habitat micromodification. By analyzing the intestinal microbiota and the proportional composition of ingested plant types across different stages of habitat micromodification, we observed that although the primary dietary components of Père David’s deer changed significantly among the pre-change group, under-change group, and post-change group, the structural composition of their gut microbiota remained relatively stable. This suggests that despite variations in food types, the core nutritional components (e.g., fiber proportions) likely remained consistent, reducing the necessity for microbiota reorganization. The core bacterial species within the Père David’s deer’s gut possess diverse metabolic pathways, enabling adaptation to different dietary substrates without requiring major structural adjustments to the microbial community. Additionally, the multi-chambered stomach of Père David’s deer prolongs food retention time, enhancing fiber degradation efficiency and stabilizing microbial metabolic activity. In future research, we will select more sensitive methods such as DNA macrobarcoding technology for dietary analysis and strive to establish a comprehensive local database of potential food sources.

Our findings carry direct implications for conservation practice. The observed resilience of the gut microbiota suggests that Père David’s deer populations possess a physiological buffer against moderate, managed habitat changes, supporting the feasibility of well-designed wetland restoration projects in their habitats. For conservation managers, this underscores that non-invasive monitoring of diet and gut microbiota can serve as a valuable tool to assess animal health and adaptation during and after habitat interventions. Furthermore, the principle of leveraging an animal’s dietary plasticity alongside a stable microbial core could inform reintroduction strategies for other endangered ruminants. Selecting release sites with diverse, fibrous vegetation and employing fecal monitoring could help evaluate pre- and post-release adaptation, increasing the likelihood of reintroduction success. In future research, we will employ more sensitive methods such as DNA metabarcoding for dietary analysis and strive to establish a comprehensive local database of potential food sources to further refine these management tools.

## Figures and Tables

**Table 1:** Relative density (RD%) of various edible plants of Père David’s deer in each group.

Taxonomic Status			Pre-Change Group RD%	Under-Change Group RD%	Post-Change Group RD%
Family	Genus	Species Name			
Poaceae	*Phragmites*	*Phragmites australis*	16.34%	10.85%	16.74%
Poaceae	*Eleusine*	*Eleusine indica*	1.39%	2.66%	3.63%
Poaceae	*Setaria*	*Setaria viridis*	5.77%	4.17%	2.76%
Poaceae	*Chloris*	*Lysimachia barystachys*	1.52%	0.55%	1.58%
Poaceae	*Pennisetum*	*Pennisetum alopecuroides*	0.97%	2.47%	0.47%
Poaceae	*Digitaria*	*Digitaria sanguinalis*	0.47%	-	-
Poaceae	*Echinochloa*	*Echinochloa crusgalli*	33.76%	4.25%	2.11%
Malvaceae	*Abutilon*	*Abutilon theophrasti*	2.45%	1.09%	0.22%
Asteraceae	*Ixeris*	*Ixeris polycephala*	-	3.11%	-
Asteraceae	*Cirsium*	*Cirsium setosum*	1.03%	1.53%	2.36%
Asteraceae	*Artemisia*	*Artemisia caruifolia*	-	-	0.22%
Asteraceae	*Cosmos*	*Cosmos bipinnatus*	0.52%	0.82%	0.73%
Asteraceae	*Bidens*	*Bidens pilosa*	0.53%	2.06%	0.51%
Chenopodiaceae	*Kochia*	*Kochia* *scoparia*	3.95%	0.57%	0.48%
Chenopodiaceae	*Suaeda*	*Suaeda glauca*	0.64%	1.05%	0.21%
Chenopodiaceae	*Chenopodium*	*Chenopodium album*	1.87%	2.64%	5.31%
Polygonaceae	*Polygonum*	*Polygonum aviculare*	0.15%	0.80%	3.19%
Polygonaceae	*Polygonum*	*Polygonum orientale*	1.03%	1.63%	2.88%
Gentianaceae	*Nymphoides*	*Nymphoides peltata*	0.20%	2.50%	-
Asclepiadaceae	*Cynanchum*	*Cynanchum chinense*	5.30%	7.19%	8.21%
Portulacaceae	*Portulaca*	*Portulaca oleracea*	0.16%	0.21%	-
Solanaceae	*Solanum*	*Solanum nigrum*	1.03%	2.32%	2.47%
Cannabaceae	*Humulus*	*Humulus scandens*	4.58%	11.23%	6.38%
Cyperaceae	*Bolboschoenus*	*Bolboschoenus yagara*	2.83%	2.04%	0.48%
Typhaceae	*Typha*	*Typha orientalis*	0.98%	0.99%	1.27%
Euphorbiaceae	*Acalypha*	*Acalypha australis*	5.37%	0.36%	0.98%
Convolvulaceae	*Ipomoea*	*Ipomoea purpurea*	3.62%	30.22%	7.18%
Nelumbonaceae	*Nelumbo*	*Nelumbo nucifera*	0.15%	2.45%	0.89%
Rubiaceae	*Rubia*	*Rubia cordifolia*	1.99%	-	0.21%
Haloragaceae	*Myriophyllum*	*Myriophyllum spicatum*	0.35%	-	-
Celastraceae	*Euonymus*	*Euonymus japonicus*	-	-	26.77%
Ulmaceae	*Ulmus*	*Ulmus pumila*	1.02%	-	1.55%
Rosaceae	*Prunus*	*Prunus persica*	-	0.22%	0.21%

## Data Availability

The original data presented in the study are openly available in NCBI at https://www.ncbi.nlm.nih.gov/bioproject/?term=+PRJNA1284050, reference number PRJNA1284050.

## Supplementary Materials

The following supporting information can be downloaded at https://www.mdpi.com/article/10.3390/microorganisms14010155/s1: Figure S1: Shannon dilution curve, Figure S2: Venn diagram of the phylum and genus levels of the intestinal microbiota (A: phylum level, B: genus level), Figure S3: Species composition diagram of intestinal microbiota (A: class level, B: Order level, C: family level), Figure S4: LEfse multi-level species difference discriminant analysis, Figure S5: Function heatmap diagram of KEGG pathway level 1, Figure S6: Function heatmap diagram of KEGG pathway level 2, Figure S7: Function heatmap diagram of KEGG pathway level 3; Table S1: The top 5 phyla and genera in terms of proportion, Table S2: Alpha diversity among three groups.
