# Supplementary material for: How Habitat Micromodification Influences Gut Microbiota and Diet Composition of Reintroduced Species: Evidence from Endangered Père David’s Deer
Source: Microorganisms. 2026 Jan 10;14(1):155. doi: 10.3390/microorganisms14010155 (PMC12844245; doi:10.3390/microorganisms14010155)
Supplement: Supplementary file 1 [file microorganisms-14-00155-s001.zip › microorganisms-4024832-supplementary.pdf]

Supplementary Table S1. The top 5 phyla and genera in terms of proportion

Group name	Phylum	Genus
pre-change group	Firmicutes (78.37%)	*Christensenellaceae_R-7_group* (11.81%)
	Bacteroidota (19.21%)	*UCG-005* (11.50%)
	Actinobacteriota (0.69%)	*Rikenellaceae_RC9_gut_group* (6.03%)
	Verrucomicrobiota (0.40%)	*norank_o__Clostridia_UCG-014* (5.90%)
	Spirochaetota (0.45%)	*norank_f__UCG-010* (5.46%)
under-change group	Firmicutes (81.38%)	*UCG-005* (17.57%)
	Bacteroidota (14.83%)	*Christensenellaceae_R-7_group* (10.48%)
	Actinobacteriota (2.29%)	*Bacillus* (8.01%)
	Verrucomicrobia (0.54%)	*norank_f__Eubacterium_coprostanoligenes_group* (6.50%)
	Fibrobacterota (0.26%)	*Monoglobus* (4.39%)
post-change group	Firmicutes (75.42%)	*UCG-005* (12.02%)
	Bacteroidota (19.35%)	*norank_o__Clostridia_UCG-014* (9.66%)
	Actinobacteriota (2.74%)	*Christensenellaceae_R-7_group* (9.31%)
	Verrucomicrobiota (0.73%)	*norank_f__Eubacterium_coprostanoligenes_group* (8.76%)
	Spirochaetota (0.62%)	*Rikenellaceae_RC9_gut_group* (5.24%)

Supplementary Table S2. Alpha Diversity among three groups

Group name	Chao index	Ace index	Simpson index	Shannon index
pre-change group	1520.4	1529.1	0.017066	5.2912
under-change group	1083.4	1954.5	0.042798	5.2071
post-change group	1596.8	1599.7	0.01703	5.3488

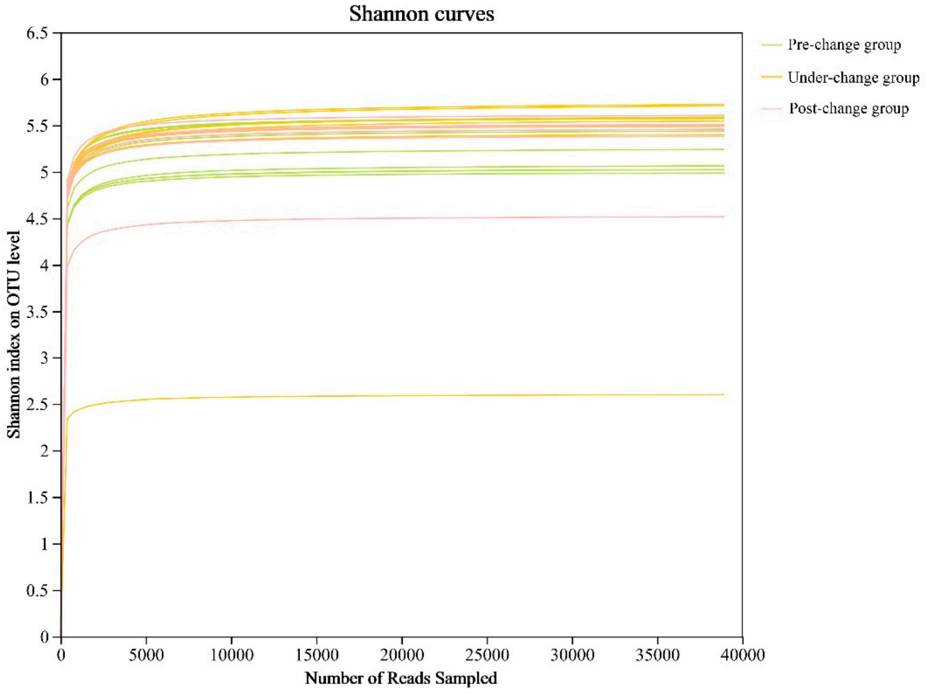

Supplementary Figure S1. Shannon dilution curve

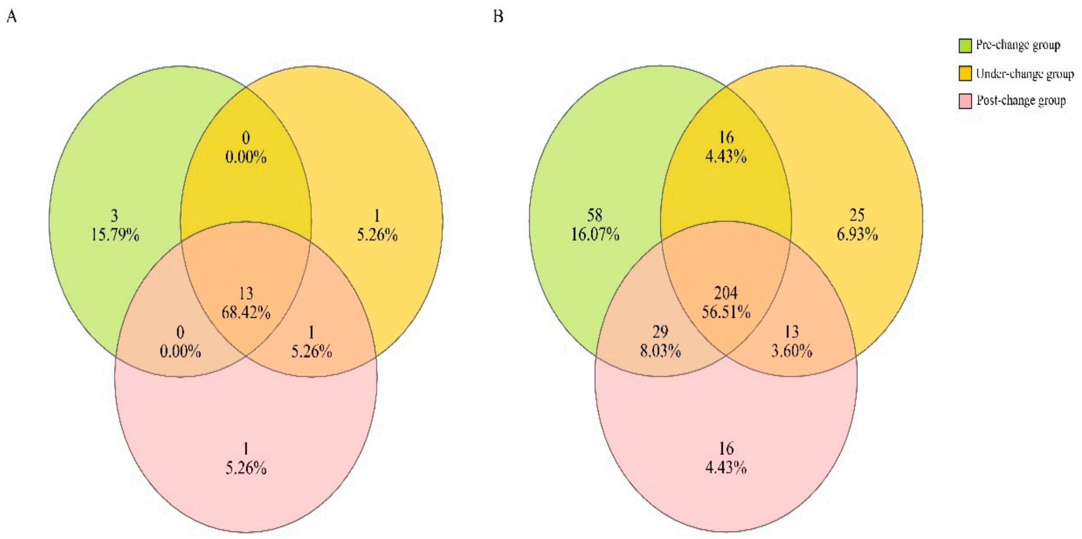

Supplementary Figure S2. Venn diagram of the phylum and genus levels of the intestinal microbiota (A: phylum level, B: genus level)

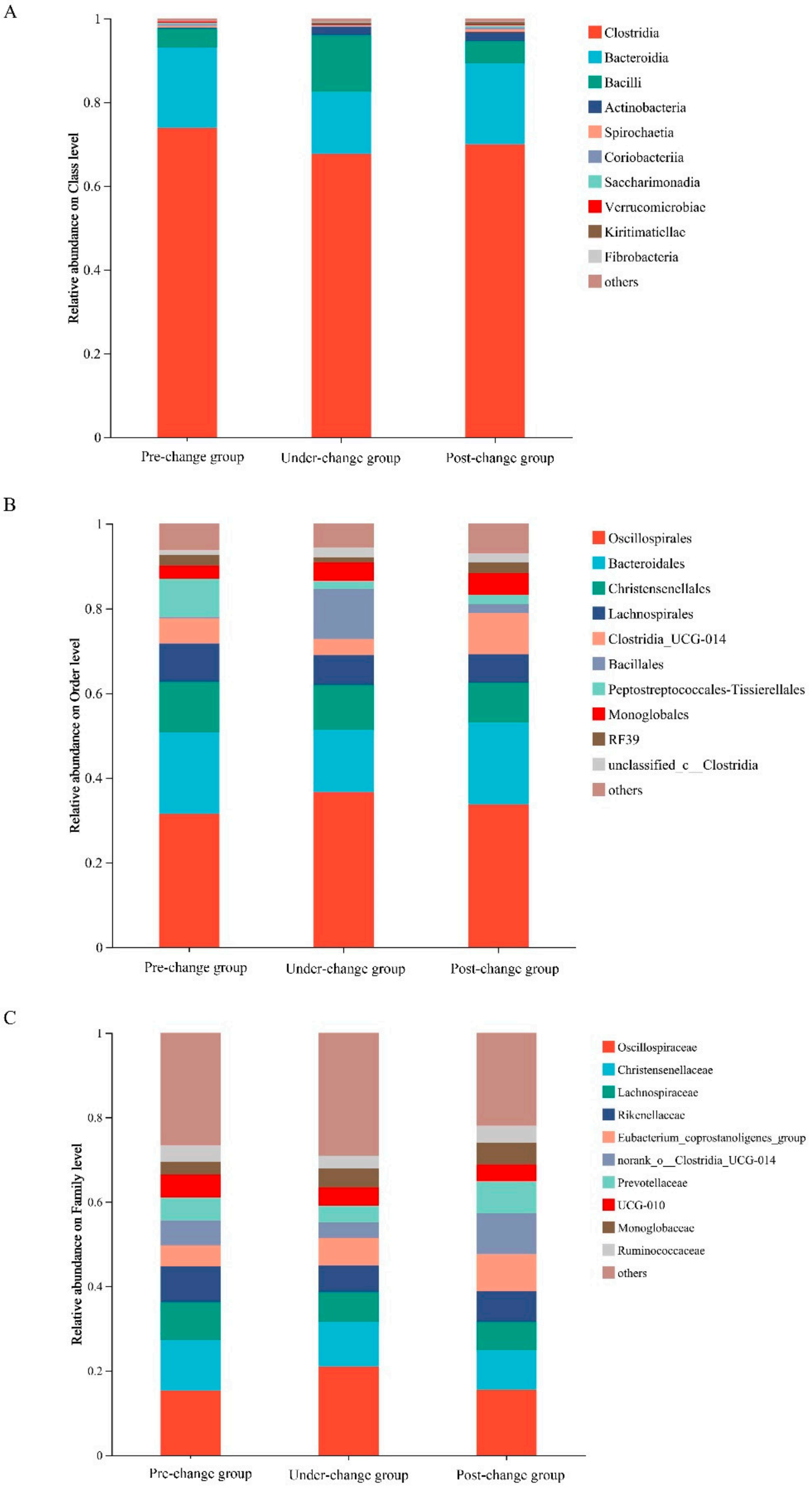

Supplementary Figure S3. Species composition diagram of intestinal microbiota (A: class level, B: Order level, C: family level)

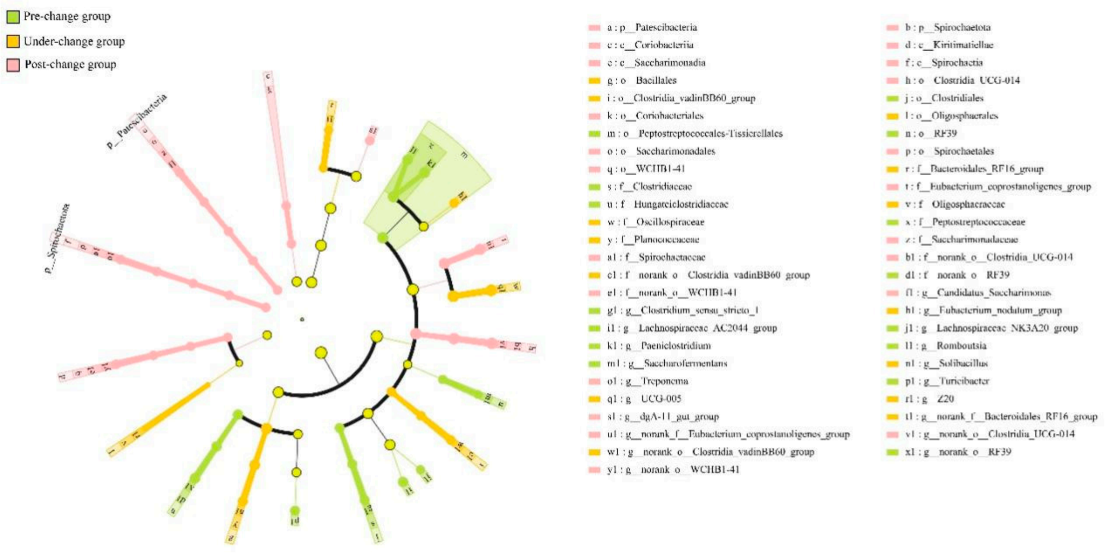

Supplementary Figure S4. LEfse multi-level species difference discriminant analysis

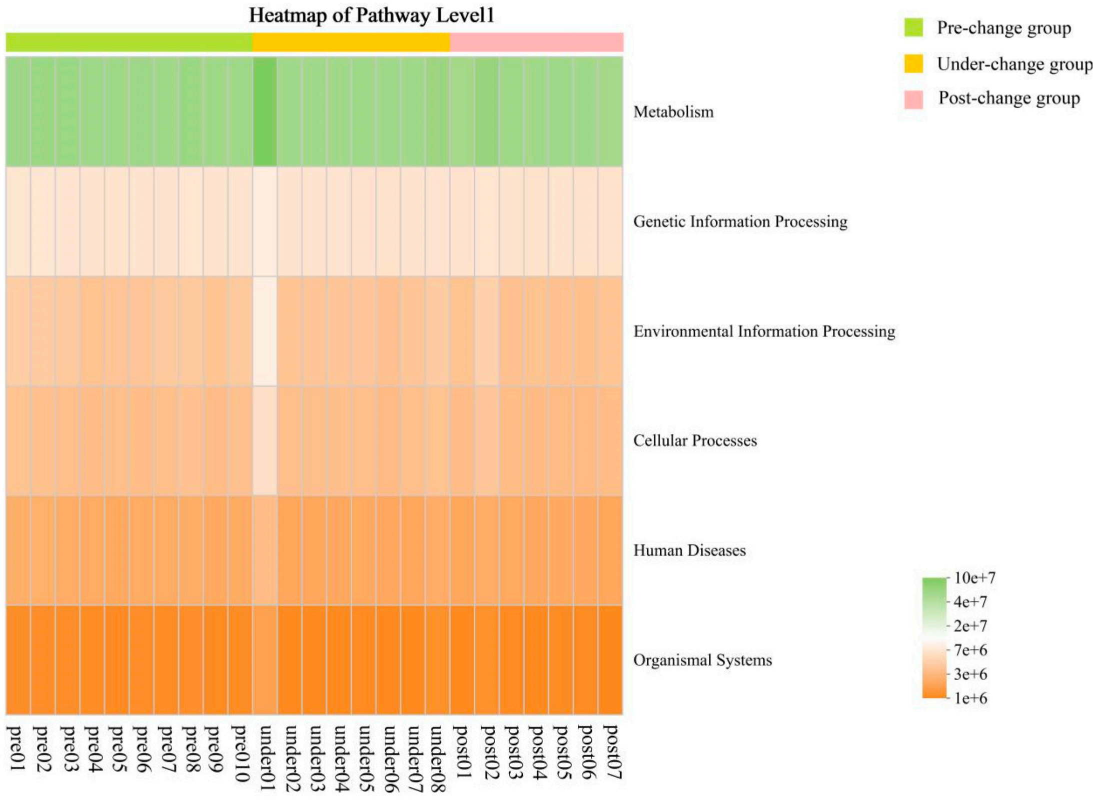

Supplementary Figure S5. Function Heatmap diagram of KEGG pathway level 1

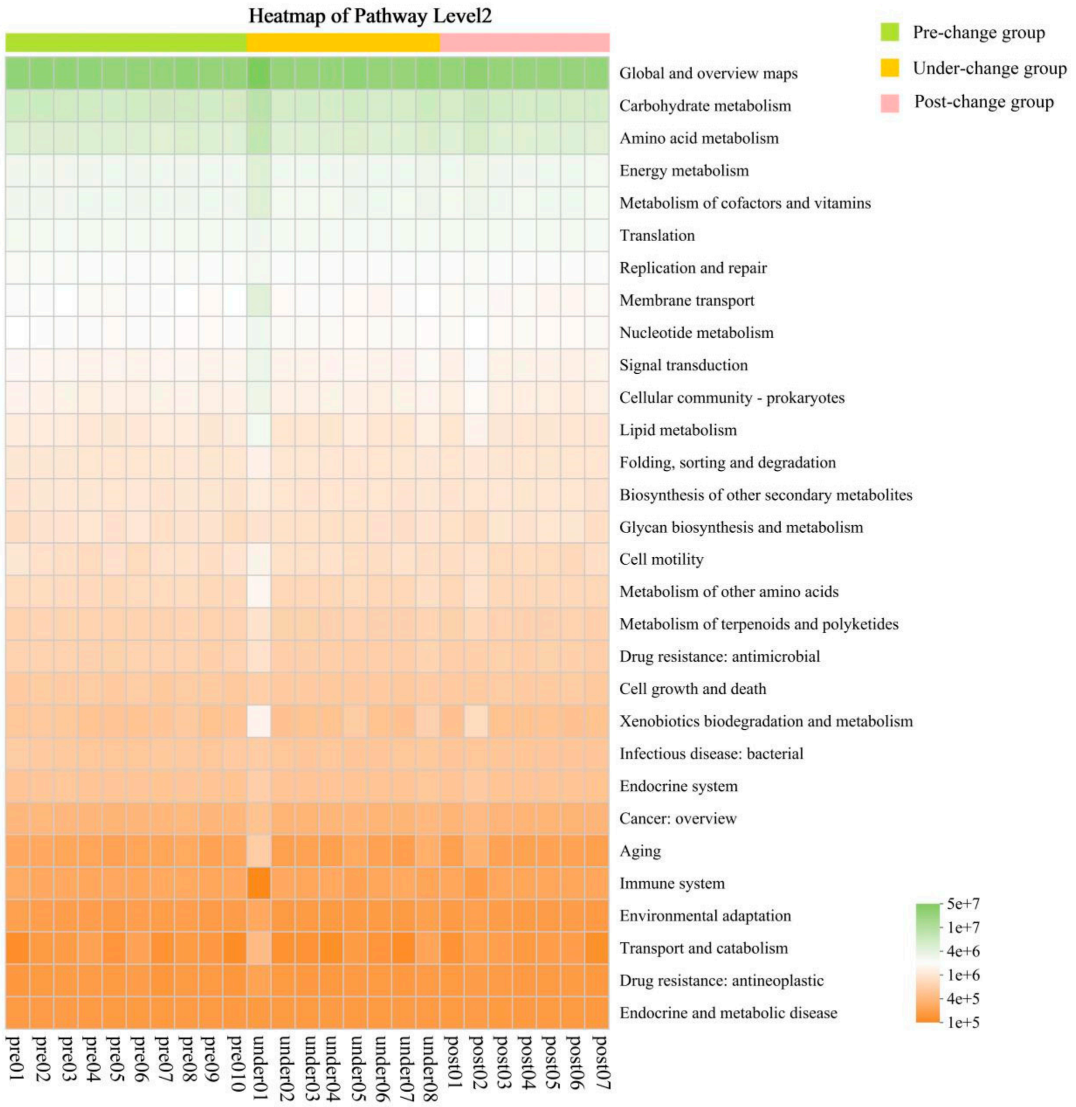

Supplementary Figure S6. Function Heatmap diagram of KEGG pathway level 2

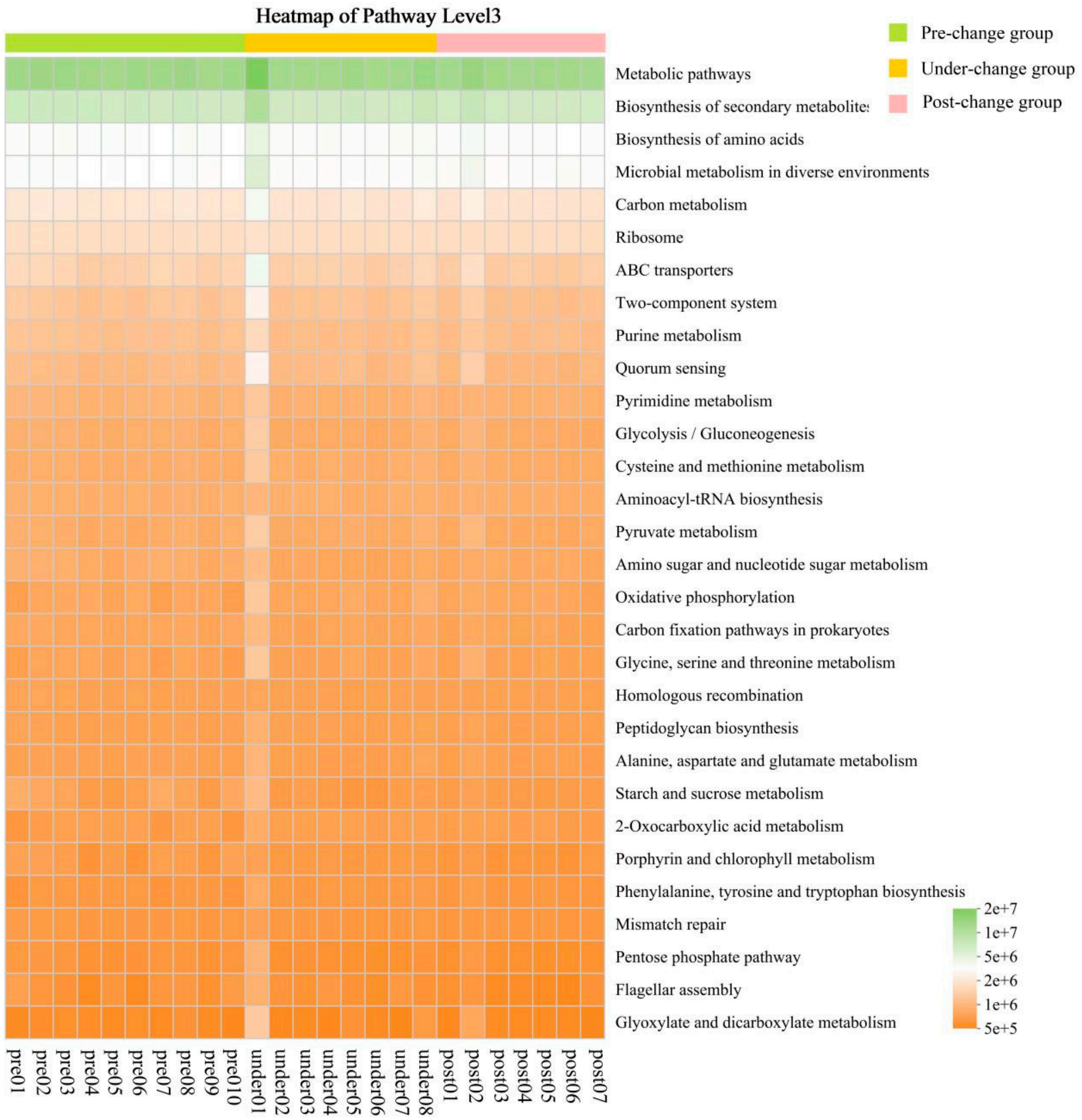

Supplementary Figure S7. Function Heatmap diagram of KEGG pathway level 3
